# International changes in end-of-life practices over time: a systematic review

**DOI:** 10.1186/s12913-016-1749-z

**Published:** 2016-10-03

**Authors:** Yi-Sheng Chao, Antoine Boivin, Isabelle Marcoux, Geneviève Garnon, Nicholas Mays, Pascale Lehoux, Marie-Claude Prémont, Evert van Leeuwen, Raynald Pineault, Advisory Committee, Canadian Medical Association, Jeff Blackmer, College of Family Physicians of Canada, Marie-Dominique Beaulieu, Bill Sullivan, Canadian Bar Association, Pierre Deschamps, Bernard Grenier, Ann Soden, Robert Delorme, Ministère de la santé et des services sociaux du Québec, Jean Rodrigue, Jeanine Auger, Louis Dufresne, Réseau des soins palliatifs du Québec, Justine Farley, Danielle Drouin, Commissaire à la santé et au bien-être du Québec, Ghislaine de Langavant

**Affiliations:** 1 University of Montreal Hospital Centre Research Centre (CRCHUM), Montreal, Canada; 2 Institut de recherche en santé publique de l’Université de Montréal, Montréal, Canada; 3 Interdisciplinary School of Health Science, University of Ottawa, Ottawa, Canada; 4 Université de Sherbrooke, Sherbrooke, Canada; 5 Department of Health Services Research and Policy, London School of Hygiene and Tropical Medicine, London, UK; 6 Département d’administration de la santé, Université de Montréal, Montreal, Canada; 7 École nationale d’administration publique, Montreal, Canada; 8 Scientific Institute for Quality of Healthcare, Radboud University Nijmegen, Nijmegen, The Netherlands; 9 Département de santé publique de Montréal, Institut National de Santé Publique du Québec, Montreal, Canada

**Keywords:** End-of-life practice, Treatment withdrawal, Aid in dying, Assisted suicide, Euthanasia

## Abstract

**Background:**

End-of-life policies are hotly debated in many countries, with international evidence frequently used to support or oppose legal reforms. Existing reviews are limited by their focus on specific practices or selected jurisdictions. The objective is to review international time trends in end-of-life practices.

**Methods:**

We conducted a systematic review of empirical studies on medical end-of-life practices, including treatment withdrawal, the use of drugs for symptom management, and the intentional use of lethal drugs. A search strategy was conducted in MEDLINE, EMBASE, Web of Science, Sociological Abstracts, PAIS International, Worldwide Political Science Abstracts, International Bibliography of the Social Sciences and CINAHL. We included studies that described physicians’ actual practices and estimated annual frequency at the jurisdictional level. End-of-life practice frequencies were analyzed for variations over time, using logit regression.

**Results:**

Among 8183 references, 39 jurisdiction-wide surveys conducted between 1990 and 2010 were identified. Of those, 22 surveys used sufficiently similar research methods to allow further statistical analysis. Significant differences were found across surveys in the frequency of treatment withdrawal, use of opiates or sedatives and the intentional use of lethal drugs (*X*^*2*^ > 1000, *p* < 0.001 for all). Regression analyses showed increased use of opiates and sedatives over time (*p* < 0.001), which could reflect more intense symptom management at the end of life, or increase in these drugs to intentionally cause patients’ death.

**Conclusion:**

The use of opiates and sedatives appears to have significantly increased over time between 1990 and 2010. Better distinction between practices with different legal status is required to properly interpret the policy significance of these changes. Research on the effects of public policies should take a comprehensive look at trends in end-of-life practice patterns and their associations with policy changes.

## Background

End-of-life policies are hotly debated in many countries, with international evidence used to support or oppose legal reforms [1–3]. For example, recent policy proposals on the legalization of “medical aid in dying”, “physician-assisted suicide” and “euthanasia” in Canada, the United Kingdom, and France have made frequent references to empirical research from other countries [4, 5].

A scoping review highlighted the need to take a comprehensive look at international end-of-life practice variations, because existing reviews tended to focus on specific practices or selected countries [6]. More specifically, trends in patterns of “euthanasia” and “assisted suicide” are most frequently discussed, with less attention being put on treatment withdrawal and the use of drugs for symptom management [7–11]. This could be problematic as it limits the ability to study the effects of public policies on the full range of end-of-life practices. Also, changes in a single jurisdiction might reflect global trends in end-of-life practices, rather than domestic patterns.

## Objective

The goal of this research was to systematically review international time trends in end-of-life practices.

## Methods

### Design

**Fig. 1:**
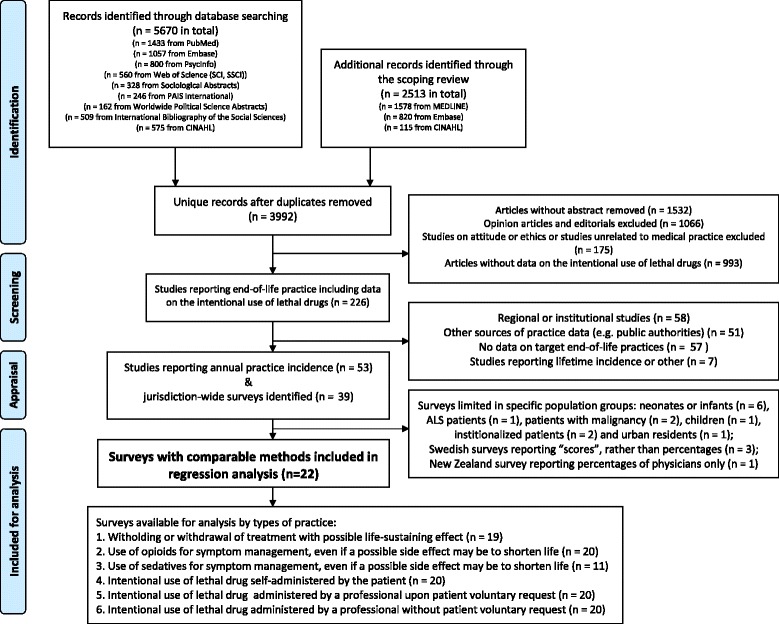
Flowchart of study inclusion and exclusion

We conducted a systematic review of empirical studies on medical end-of-life practices (see Fig. 1 for the flowchart).

### Definitions and classification of end-of-life practices

Definitions and labelling of medical end-of-life practices vary and no consensus exists on terminology at the international level. For example, definitions of “euthanasia” have evolved over time and across jurisdictions [12, 13]. For these reasons, we developed a descriptive classification of medical end-of-life practices. The classification is based on observable medical behaviors that can be studied empirically, and distinguishes practices with different legal status across jurisdictions, including:

1. Withdrawing or withholding of treatments that have the potential to prolong life;
2. Use of drugs for symptom management even if an unintended side-effect may be to shorten life;
3. Intentional use of a lethal drug.

The classification further distinguishes whether end-of-life practices are carried out: a) with a voluntary and informed request made by the patient; b) with a voluntary advanced directive made by a previously competent patient, c) with a substitute request by the proxy decision-maker of an incompetent patient; or d) without a patient or substitute request. We also distinguish whether lethal drugs are administered by patients or by someone else.

Testing of this classification in a scoping review allowed for the translation of different end-of-life practice definitions into comparable categories, despite variations in labeling and definitions used in the original studies [6].

### Inclusion criteria

To be included, articles written in English or French needed to: 1) report on physicians’ actual end-of-life practices (rather than their opinions and attitudes); 2) include data on treatment withholding/withdrawal, use of drugs for symptom management, and the intentional use of lethal drugs; 3) allow estimates of annual practice frequency at the jurisdictional level. We excluded articles limited to specific populations (e.g. neonates) or practice settings (e.g. nursing homes). We also excluded articles using physicians’ self-reporting of end-of-life practices to public regulatory authorities (e.g. euthanasia review committees) as this data source would not allow comparisons across jurisdictions with different legislative frameworks and would not provide information on some categories of practices included in our classification (e.g. intentional use of lethal drugs without patient request).

### Search strategy

The literature databases searched were: MEDLINE, Embase, PsycInfo, Web of Science (SCI, SSCI), Sociological Abstracts, EMBASE, PAIS International, Worldwide Political Science Abstracts, International Bibliography of the Social Sciences and CINAHL. Search terms are listed in Appendix 1. The literature was searched in April 2014 and were pooled with articles identified in a previous scoping review that also included grey literature and online publications [6].

### Data screening and extraction

Two reviewers separately screened each article and any discrepancies regarding the inclusion or exclusion of articles were resolved in team meetings. The information and statistics on these articles were extracted by one of two assistants and two review authors, verified by one of the review authors (GG or YSC), and entered in a FileMaker Pro v. 13 database developed with an information technology specialist. The items extracted from articles included year of publication, authors, title, abstract, source database, the types of end-of-life practice studied, funding sources, types of study (qualitative, quantitative or mixed), characteristics of study participants, and affiliations of first authors. When the same survey was reported in multiple articles, an index article was used to extract information on data collection methods (including duration, population groups and region of study), response rates, selective reporting for outcomes, details in statistical procedures, total numbers of deaths and summary percentages of end-of-life practices in each jurisdiction.

### Comparability of study methods and risk of bias

The objectives for assessing the risk of bias were to 1) identify the risk of bias in estimated frequencies; 2) understand the heterogeneity of study methods to identify a subset of comparable studies using similar reference populations and outcome measures. We used quality appraisal criteria designed for systematic reviews [14, 15] and observational studies [16, 17]. The assessment criteria were: background information (population coverage, number of studied deaths, total number of deaths in the jurisdiction) [18]; basic survey information (article types, study design, author affiliations) [16]; design-specific assessment (statistical, conflict of interest, response rate, frequency of measurement, sample sizes, methods to adjust for selection bias) [19–21]; and practice-related assessment (methods for measuring outcome variables and selective outcome reporting) [22]. After reviewing the included surveys, the comparability of study design and statistical methods were assessed in the team meetings. Surveys using comparable methods (population characteristics, questionnaire types, statistical methods, and reporting methods) were retained for regression analysis.

### Data analysis

The main outcome measure was the annual frequency of end-of-life practices as a percentage (%) of all deaths [7]. We further collected the 95 % confidence intervals (CIs) of the weighted frequencies or the nominators and denominators of the reported frequencies.

The extracted data were analyzed with R programming languange and RStudio (version 0.98.1091, RStudio Inc.). The surveys that adopted multiple-stage sampling usually provided the mean percentages of end-of-life practice with 95 % CIs. The comparisons of the percentages of end-of-life practices across jurisdictions were carried out using Chi-squared tests. If Chi-squared tests were not appropriate, simulation was used [23].

The relationships between the frequencies of end-of-life practices and time (year of data collection) were assumed to be linear and were analyzed with logit regression. The null hypotheses were that there were no associations between the frequencies and time (regression coefficients of time equal to zero). Jurisdictions or survey types that were found to be significantly associated with different levels of end-of-life practice frequencies [7] were adjusted in models that included all eligible surveys. Due to the variability originating from stratified sampling and adjustment for non-response, the 95 % CIs of reported frequencies were taken into account using simulations. The mean percentages were used to draw the regression lines in the graphs. The magnitudes of change in end-of-life practices from all included surveys were estimated with the differences in the predicted frequencies of the Dutch death certificate surveys from 1990 (or 2001 for use of sedatives for symptom management) to 2010 based on the regression models that included all surveys, since the Dutch surveys were the only ones to have been implemented throughout the period. The Dutch and Belgian surveys were also analyzed with separate regression models.

## Results

Among 8183 references, 39 jurisdiction-wide surveys were identified. Of those, 22 surveys used sufficiently similar research methods to allow further statistical analysis and comparisons (Appendix 2). Response rates ranged from 40.0 to 84.0 % (median: 65.9 %). The 22 included surveys adopted or modified questionnaires that were developed in the Netherlands in the early 1990s [24] and used similar definitions of end-of-life practices, survey methods, and statistical analysis. Two main sampling strategies were used to identify responding physicians: random sampling of death certificates, and the use of physician registries. In these surveys, physicians were either interviewed individually or responded to self-administered questionnaires.

**Fig. 2:**
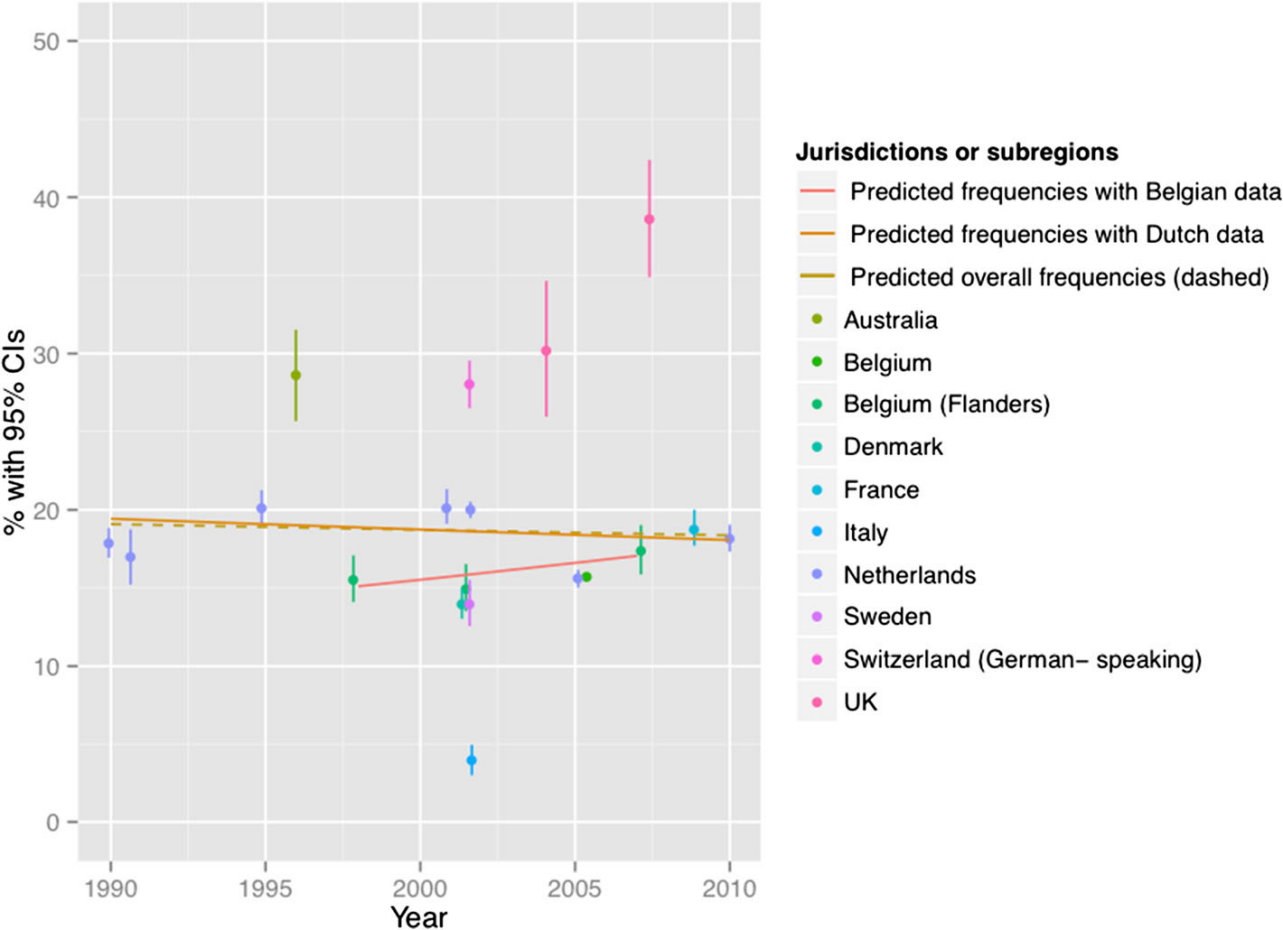
Withholding or withdrawal of treatment with the potential to prolong life. Note: CI = confidence interval; United Kingdom = UK. *X* ^*2*^ = 36833 (null hypothesis: all frequencies the same); *p* < 0.001. The coefficient of year = −0.002, *p* < 0.001 among all surveys; greater than zero suggesting positive changes from 1990 to 2010, lower suggesting the opposite. The coefficient of year = −0.0045, *p* < 0.001 among Dutch surveys only. The coefficient of year = 0.0161, *p* < 0.001 among Belgian surveys only. See Appendix 3 for regression coefficients

**Fig. 3:**
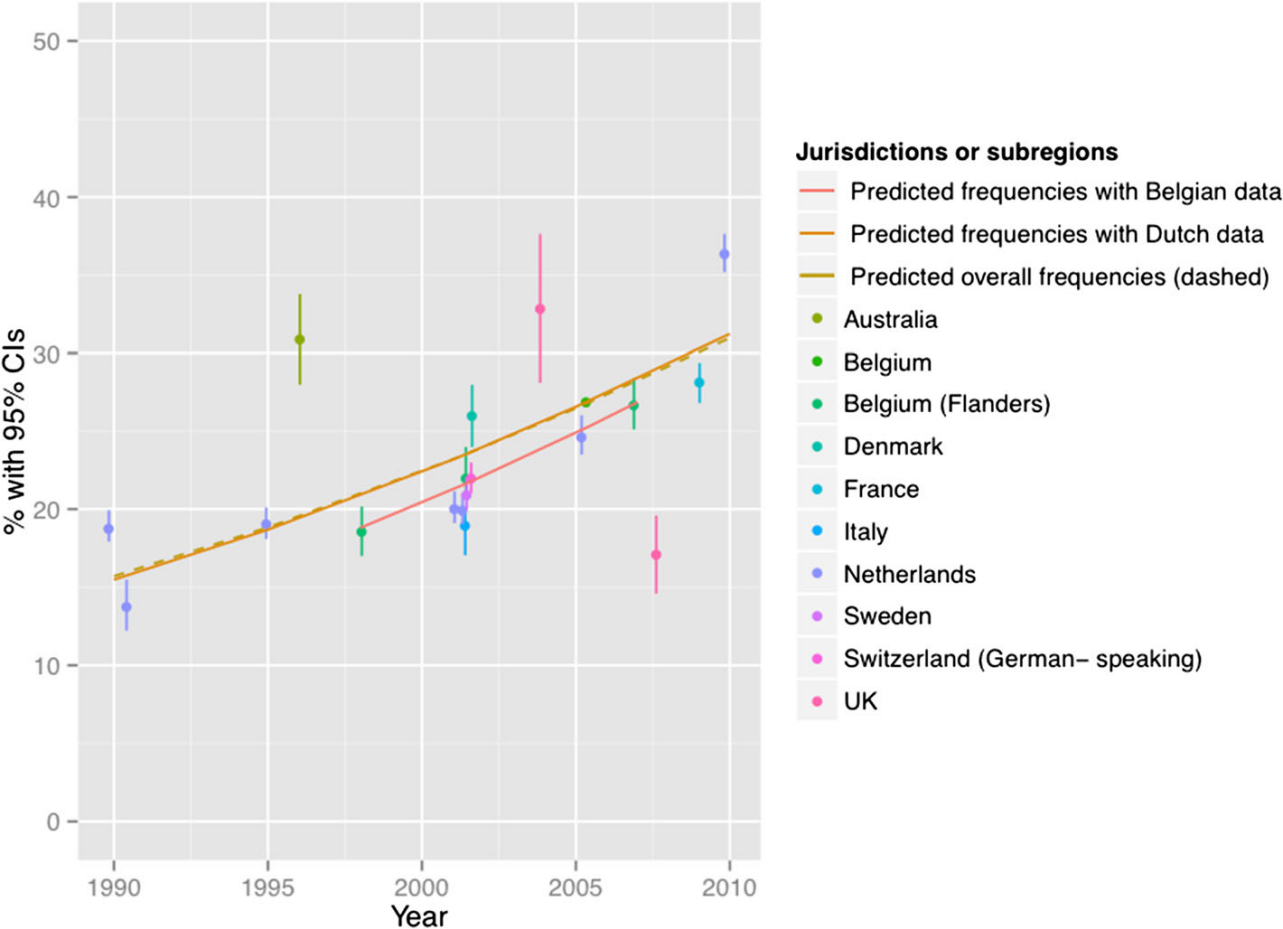
Use of opiates with possible life-shortening effects. Note: See Fig. 2 for abbreviations. *X* ^*2*^ = 37199 (null hypothesis: all frequencies the same), *p* < 0.001. Thecoefficient of year = 0.0440, *p* < 0.001 among all surveys; greater than zero suggesting positive changes from 1990 to 2010, lower suggesting the opposite). The coefficient of year = 0.0454, *p* < 0.001 among Dutch surveys. The coefficient of year = 0.0513, *p* < 0.001 among Belgian surveys. See Appendix 3 for regression coefficients

**Fig. 4:**
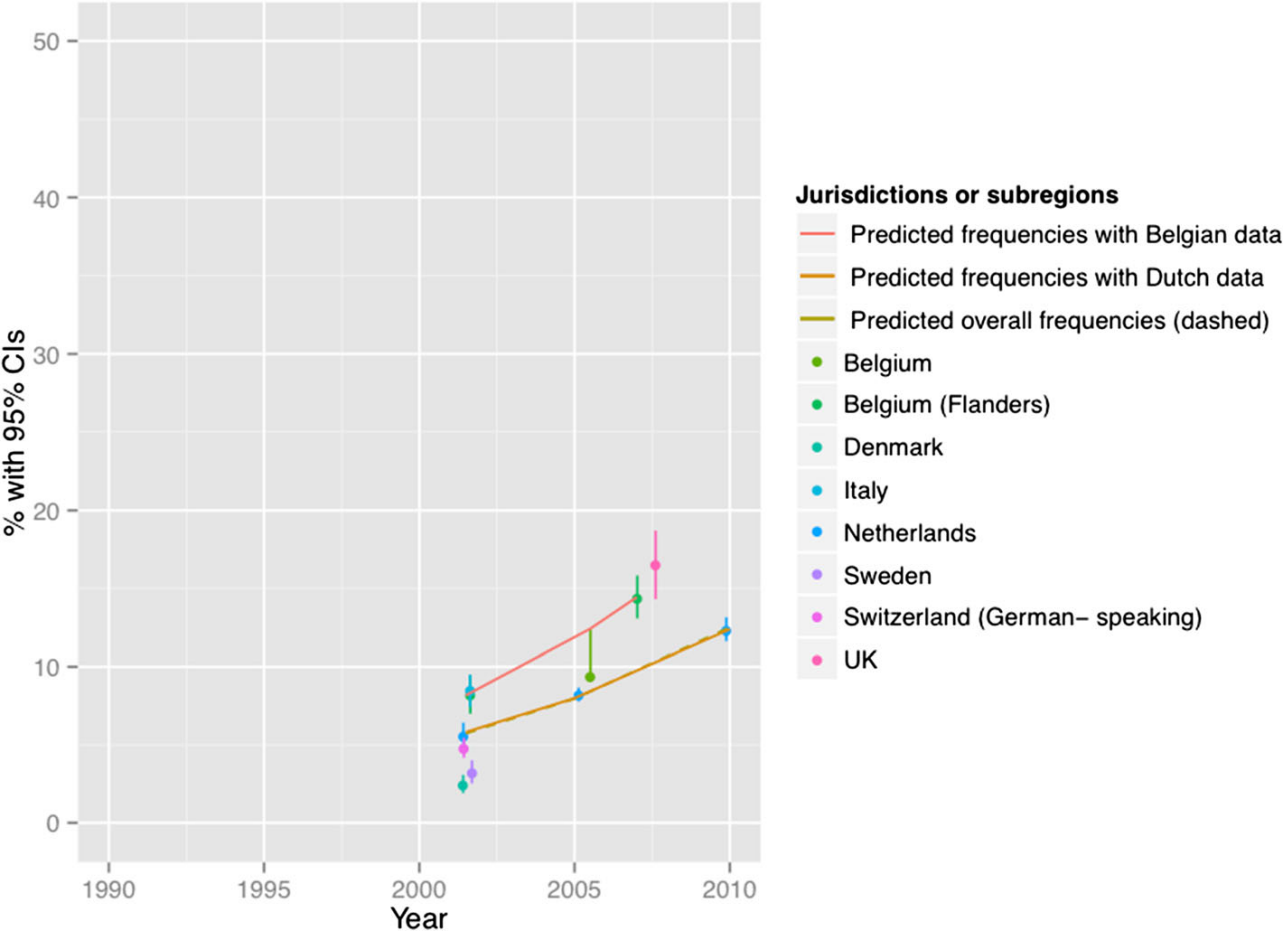
Use of sedatives with possible life-shortening effects. Note: See Fig. 2 for abbreviations. *X* ^*2*^ = 19410 (null hypothesis: all frequencies the same), *p* < 0.001. The coefficient of year = 0.1006, *p* < 0.001 among all surveys; greater than zero suggesting positive changes from 1990 to 2010, lower suggesting the opposite. The coefficient of year = 0.0974, *p* < 0.001 among Dutch surveys. The coefficient of year = 0.1162, *p* < 0.001 among Belgian surveys. See Appendix 3 for regression coefficients

**Fig. 5:**
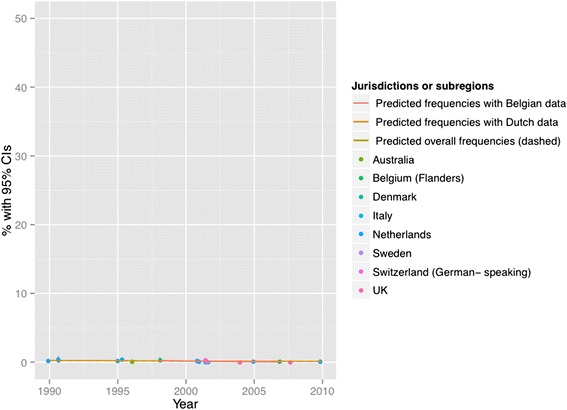
Intentional use of lethal drugs self-administered by patients. Note: See Fig. 2 for abbreviations. *X* ^*2*^ = 1716 (null hypothesis: all freqiuencies the same), *p* < 0.001. The coefficient of year = −0.0492, *p* < 0.001 among all surveys; greater than zero suggesting positive changes from 1990 to 2010, lower suggesting the opposite. The coefficient of year = −0.0442, *p* < 0.001 among Dutch surveys. The coefficient of year = − 0.2291, *p* < 0.001 among Belgian surveys. See Appendix 3 for regression coefficients

**Fig. 6:**
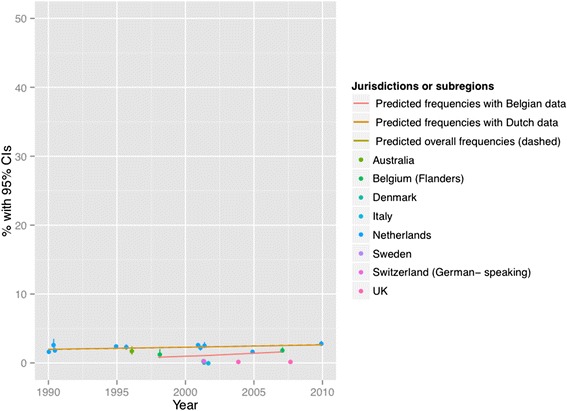
Intentional use of lethal drugs administered by professionals with patient request. Note: See Fig. 2 for abbreviations. *X* ^*2*^ = 8678 (null hypothesis: all frequencies the same), *p* < 0.001 (simulated). The coefficient of year = 0.0159, *p* < 0.001 among all surveys; greater than zero suggesting positive changes from 1990 to 2010, lower suggesting the opposite). The coefficient of year = 0.0142, *p* < 0.001 among Dutch surveys. The coefficient of year = 0.0730, *p* < 0.001 among Belgian surveys. See Appendix 3 for regression coefficients

**Fig. 7:**
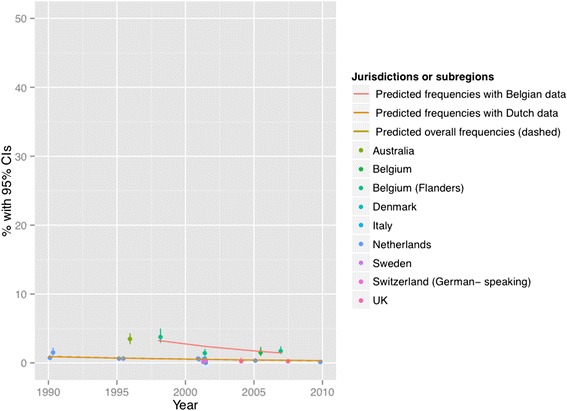
Intentional use of lethal drugs administered by professionals without patient request. Note: See Fig. 2 for abbreviations. *X* ^*2*^ = 17372 (null hypothesis: all frequencies the same), *p* < 0.001 (simulated). The coefficient of year = −0.0574, *p* < 0.001 among all surveys; greater than zero suggesting positive changes from 1990 to 2010, lower suggesting the opposite). The coefficient of year = −0.0496, *p* < 0.001 among Dutch surveys. The coefficient of year = −0.0924, *p* < 0.001 among Belgian surveys. See Appendix 3 for regression coefficients

Figures 2, 3, 4, 5, 6 and 7 present end-of-life practice frequencies by year. The original studies did not allow to fully distinguishing end-of-life practices according to their legal status, as per our descriptive classification (Table 1). For example, original studies did not distinguish treatment withdrawal with or without voluntary patient request. Also, the use of opiates and sedatives did not distinguish whether these drugs were adjusted to symptom management or used with the intention to cause death.

**Table 1:** Relationship between systematic review classification and original studies

Figure number	Systematic review classification	Example of labelling and definitions used in original study
2	Witholding or withdrawal of treatment with the potential to prolong life	« Non-treatment decisions »: the withholding or withdrawal of treatment in situations where the treatment would probably have prolonged life [24]; withhold or withdraw medical treatment while taking into account the possibility or certainty that this would hasten the patient’s death or with the explicit intention of hastening the patient’s death [31]; withheld or withdrawn medical treatment while taking into account the possible hastening of death (only for cases in which there was no single most explicit intention, the administration of drugs prevailed over the withholding or withdrawing of treatment) [36]. *Note* : *Inconsistently report whether drugs were administered with or without patient voluntary request*.
3	Use of opiates with possible life-shortening effects	« Alleviation of pain and symptoms » : alleviation of pain and symptoms with opioids in such dosages that the patient’s life might have been shortened [24]; intensify the alleviation of pain and suffering while taking into account the possibility or certainty that this would hasten the patient’s death or partly with the intention of hastening the patient’s death [31]. *Note* : *includes cases where opioids were used with the intention to hasten death. Inconsistently report whether drugs were administered with or without patient voluntary request*.
4	Use of sedatives with possible life-shortening effects	« Continuous deep sedation »: deeply and continuously sedated until death [36]; the patient receive drugs, such as barbiturates or benzodiazepines, to keep him/her continuously in deep sedation or coma until death [26]. *Note* : *does not distinguish whether sedatives were used with or without the intention to hasten death. Inconsistently report whether drugs were administered with or without patient voluntary request*.
5	Intentional use of lethal drugs self-administered by patients	« Assisted suicide »: the prescription or supply of drugs by a physician with the explicit intention of shortening life, when the drug is administered by patients [24, 31]. *Note* : *does not include the use of drugs with the* « *partial* » *intention to cause death*.
6	Intentional use of lethal drugs administered by professionals with patient request	« Euthanasia »: the prescription, supply or administration of drugs with the explicit intention of shortening life, when the dru gis administered by professionals [24]; death as the result of the administration, supply, or prescription of drugs with the explicit intention of hastening the patient’s death (administered by professionals) [31]. *Note* : *does not include the use of drugs with the* « *partial* » *intention to cause death*.
7	Intentional use of lethal drugs administered by professionals without patient request	« Termination of life without a patient request »: the prescription, supply or administration of drugs with the explicit intention of shortening life (without patient explicit request) [24]; death as the result of the administration, supply, or prescription of drugs with the explicit intention of hastening the patient’s death (without patient explicit request) [31]. *Note* : *does not include the use of drugs with the* « *partial* » *intention to cause death*.

Treatment withholding/withdrawal and the use of drugs for symptom management were the most frequent end-of-life practices. The intentional use of lethal drugs was less frequent, with frequency consistently below 5 % of all deaths.

The total predicted percentages of death in which physicians reported having made an end-of-life decision increased from 38.0 to 64.9 % between 1990 and 2010. Significant changes were found in the frequency of all end-of-life practices over time (*p* < 0.001 for all). The magnitude of change in the predicted frequencies of end-of-life practices was greater for the use of opioids (+15.27 % in annual practice frequency between 1990 to 2010) and for the use of sedatives with possible life-shortening effects (+6.78 %). Changes in frequencies were smaller for treatment withholding or withdrawal (−0.73 %), and for the intentional use of lethal drugs self-administered by patients (−0.17 %), administered by professionals with patient request (+0.71 %), or administered by professionals without patient request (−0.65 %). Changes in opioid use were significantly correlated with changes in: the use of sedatives, in the intentional use of lethal drugs administered by patients, and in the intentional use of lethal drugs without patient request (*p* < 0.05). Time trends estimated from Dutch or Belgian data showed the same directions of change as those predicted from all jurisdictions, except for withholding or withdrawal of treatment (*p* < 0.001).

## Discussion

### Key findings

To the best of our knowledge, this is the first systematic review to take a comprehensive look at international variations in end-of-life practices over time from 1990 to 2010. This review showed an increase in the frequency of cases where physicians reported having made a decision that may have influenced the timing of death, which is consistent with previous findings [11]. A unique contribution of this review is its documentation of a significant increase in the use of opiates and sedatives over time, as opposed to other end-of-life practices.

The way opiate and sedative use is categorized in the original studies makes it difficult to interpret these time trends, because study questionnaires do not clearly distinguish the use of opiates and sedatives with or without the intention to hasten death [24]. For example, practices labeled in original studies as “intensification of symptom alleviation” include the use of drugs with a partial intention to hasten death [25–27]. Accordingly, the observed increase in the use of opiates and sedatives could mean that physicians are becoming more prone to use these drugs for symptom management. It may also reflect increasingly using opiates and sedatives with the intention of hastening patients’ death among physicians. The observation that the use of opiates with possible life-shortening effects is negatively correlated with the intentional use of lethal drugs could support the hypothesis of a “substitution effect” between different end-of-life practices. This suggests that drugs clearly associated with an intention to hasten death (e.g. neuromuscular blockers) are being replaced by opiates and sedatives in situations where the use of lethal drug is more difficult to justify in legal terms. Alternatively, these trends could simply reflect more professional willingness and patients’ expectations to treat pain and symptoms at the end-of-life. Use of these drugs could have become more strongly embedded in clinical and social practices as more information becomes available about the beneficial use of opiates and sedatives for symptom management [28].

### Policy and research implications

This review highlights the importance of exploring the potential effects of public policies on all end-of-life practices, rather than focusing exclusively on those targeted in policy documents. For example, a number of studies have explored the relationship between “euthanasia” legalization in the Netherlands and Belgium and the use of lethal drugs with the explicit intention to hasten death [29, 30]. However, the review shows that the magnitude of predicted changes in intentional use of lethal drugs is much less than the use of opioids or sedatives with possible life-shortening effects. Focusing on small changes in the frequencies of intentional use of lethal drugs risks overlooking the greater potential impact on the use of opiates and sedatives in end-of-life care.

This review also highlights the need for better articulation between end-of-life research and policy. The current confusion in empirical research between the use of opiates and sedatives with or without the intention to hasten death makes it difficult to interpret changing patterns in the use of these drugs because practices with different legal status are grouped together [6]. Better alignment between end-of-life practice classification in policy documents and empirical research would facilitate testing of policy-relevant hypotheses about the potential impacts of different policies on end-of-life practices. Our findings also underscore the importance of international research collaborations to harmonize study methods. Such collaborations have emerged in Europe (e.g. the EURELD consortium [31]), but are less developed in North America and elsewhere, which limits the potential for international comparisons.

### Strengths and limitations

A strength of this review is the exhaustive efforts made to synthesize international evidence on a range of end-of-life practices. This approach offers a complementary perspective to previous reviews that focused on a single practice or a specific jurisdiction [7, 10, 29, 32]. The use of a descriptive classification of end-of-life practices is a novel approach to systematically review evidence on end-of-life practice, and helped deal with international variations in labeling and definitions. This descriptive classification also clarifies areas of potential confusion when practices with different legal status were grouped together in original studies. Variations due to sampling and weighting procedures were also taken into account in the statistical analysis of end-of-life practice frequency. This is an improvement upon previous reviews that did not conduct statistical tests at all, or neglected the sampling variability of reported frequencies [7].

There are several limitations in this review. We only search for studies published in English and French. The increase in opioid use may result from both the intent to manage symptoms and to hasten death and we do not have evidence to assess this relationship. We found that comparative international evidence is limited by differences in study methods, end-of-life practice definitions, data collection approaches, sampling strategies, response rates, and target patient and professional groups. A limited number of surveys used similar methods allowing comparisons across jurisdictions and over time. Nonetheless, some degree of heterogeneity remains in the included surveys. For example, the two United Kingdom studies included surveyed different physician groups and used different versions of the same questionnaire [33, 34]. Other European surveys also excluded children under one years old [31] or drew their sample from a specific region (e.g. Flanders in Belgium) [35]. As a result, some of the observed international variations may still reflect differences in study methods rather than actual differences in practice frequency. Also, only a limited number of European jurisdictions have conducted repeated surveys over time. Accordingly, regression models are largely driven by data from the Netherlands and Belgium, and observed time trends may be less generalizable to other jurisdictions. The lack of studies in other jurisdictions, such as the United States and Canada may also limit the generalizability of ther results.

## Conclusions

The use of opiates and sedatives with possible life-shortening effects appears to have significantly increased over time from 1990 to 2010. Treatment withholding/withdrawal and the use of drugs for symptom management are the most frequent end-of-life practices. The intentional use of lethal drugs is less frequent, with frequency consistently below 5 % of all deaths. Better distinction between practices with different legal status is required to properly interpret the policy significance of these changes. Research on the effects of public policies should take a comprehensive look at changing the end-of-life practice patterns, rather than focus on a limited range of practices.

## Appendix 1

**Table 2:** The search terms for each academic database

	PubMed	Embase	PsycInfo	Web of Science (SCI, SSCI)	CINAHL	International Bibliography of the Social Sciences	Sociological abstracts	PAIS International	Worldwide Political Science Abstracts
Euthanasia	Euthanasia[MAJR:NOEXP] OR Euthanasia, active[MAJR:NOEXP] OR Euthanasia, Active, Voluntary[MAJR] OR Suicide, assisted[MAJR] OR Deep sedation[MAJR] OR Euthanasia*[TI] OR Assisted suicide*[TI] OR Assisted death*[TI] OR Assisted dying[TI] OR "Aid in dying" [TI] OR "Termination of life"[TI] OR Shorten life[TI] or Shortens life[TI] or Life Shorten*[TI] or Mercy killing[TI] OR ("End of life"[TI] AND (Decision*[TI] OR Practice*[TI])) OR Lethal drug*[TI] OR Deep sedation[TI] OR Continuous sedation[TI] OR Terminal sedation[TI]	*Euthanasia/or *Active euthanasia/or *Voluntary euthanasia/OR *Assisted suicide/OR *Deep sedation/or (Euthanasia? OR (Assisted adj2 (Suicide? OR Death? OR Dying)) or "Aid in dying" OR ((Termination or Shorten*) adj2 Life) or Mercy killing OR ("End of life" adj2 (Decision? OR Practice?)) OR Lethal drug? OR ((Deep or Continuous or Terminal) adj2 Sedation)).ti	*Euthanasia/or *Assisted Suicide/or (Euthanasia? OR (Assisted adj2 (Suicide? OR Death? OR Dying)) or "Aid in dying" OR ((Termination or Shorten*) adj2 Life) or Mercy killing OR ("End of life" adj2 (Decision? OR Practice?)) OR Lethal drug? OR ((Deep or Continuous or Terminal) adj2 Sedation)).ti	TI = (Euthanasia* OR (Assisted NEAR/2 (Suicide? OR Death? OR Dying)) OR "Aid in dying" OR "Mercy killing" OR (Life NEAR/2 (Shorten* or Termination)) OR ("End of life" NEAR/2 (Decision? OR Practice?)) OR "Lethal drug*" OR (Sedation NEAR/2 (Deep OR Continuous OR Terminal)))	MM "Euthanasia" OR MM "Suicide, Assisted" OR TI (Euthanasia* OR (Assisted N2 (Suicide? OR Death? OR Dying)) OR "Aid in dying" OR "Mercy killing" OR (Life N2 (Shorten* or Termination)) OR ("End of life" N2 (Decision? OR Practice?)) OR "Lethal drug*" OR (Sedation N2 (Deep OR Continuous OR Terminal)))	TI,SU(Euthanasia* OR (Assisted NEAR/2 (Suicide* OR Death* OR Dying)) OR "Aid in dying" OR "Mercy killing" OR (Life NEAR/2 (Shorten* or Termination)) OR ("End of life" NEAR/2 (Decision* OR Practice*)) OR "Lethal drug*" OR (Sedation NEAR/2 (Deep OR Continuous OR Terminal)))	TI,SU(Euthanasia* OR (Assisted NEAR/2 (Suicide* OR Death* OR Dying)) OR "Aid in dying" OR "Mercy killing" OR (Life NEAR/2 (Shorten* or Termination)) OR ("End of life" NEAR/2 (Decision* OR Practice*)) OR "Lethal drug*" OR (Sedation NEAR/2 (Deep OR Continuous OR Terminal)))	TI,AB,SU(Euthanasia* OR (Assisted NEAR/2 (Suicide* OR Death* OR Dying)) OR "Aid in dying" OR "Mercy killing" OR (Life NEAR/2 (Shorten* or Termination)) OR ("End of life" NEAR/2 (Decision* OR Practice*)) OR "Lethal drug*" OR (Sedation NEAR/2 (Deep OR Continuous OR Terminal)))	TI,AB,SU(Euthanasia* OR (Assisted NEAR/2 (Suicide* OR Death* OR Dying)) OR "Aid in dying" OR "Mercy killing" OR (Life NEAR/2 (Shorten* or Termination)) OR ("End of life" NEAR/2 (Decision* OR Practice*)) OR "Lethal drug*" OR (Sedation NEAR/2 (Deep OR Continuous OR Terminal)))
Systematic review	Meta-analysis[PT] or ((Review[PT] or Review[TI]) AND (PubMed[TIAB] or Medline[TIAB] or Embase[TIAB] or CINAHL[TIAB] or PsycInfo[TIAB])) or Cochrane Database Syst Rev[TA] or Meta-Analysis[TI] or Metaanalys*[TI] or Systematic[TI]	Meta analysis/OR Systematic review/or ((Review/or Review.ti) AND (PubMed or Medline or Embase or CINAHL or PsycInfo).ab) or Cochrane.jw or (Meta-Analysis or Metaanalys* or Systematic).ti	(Meta analysis or Systematic review).id,md or ((Literature review/or Literature Review.id,md or Review.ti) AND (PubMed or Medline or Embase or CINAHL).ab) or Cochrane.jw or (Meta-Analysis or Metaanalys* or Systematic).ti	TS = (PubMed or Medline or Embase or CINAHL or PsycInfo) AND DOCUMENT TYPES: (Review) OR TI = ("Meta Analysis" OR Systematic OR Metaanalys*)	MH "Meta-Analysis" or MH "Systematic review" OR PT Systematic review or ((PT Review or TI Review) AND AB (PubMed or Medline or Embase or CINAHL or PsycInfo)) or JT Cochrane or TI (Meta-Analysis or Metaanalys* or Systematic)				
Empirical studies	Comparative study[PT] or Observational Study[PT] OR Cohort studies[MH] OR Cross-Sectional Studies[MH] OR Cross Cultural Comparison[MH] or Anthropology, Cultural[MH:NOEXP] OR Empirical research[MH] OR "Interviews as Topic"[MH] OR Questionnaires[MH] OR "Physician's Practice Patterns"[MH] OR "Statistics and numerical data"[SH] OR Trends[SH] OR "Before and after"[TI] OR Cohort[TI] OR Comparison[TI] OR Comparative[TI] OR Cross sectional[TI] OR Cultural anthropolog*[TI] OR Descriptive[TI] OR Empirical[TI] OR Ethnograph*[TI] OR Focus group*[TI] OR Follow back[TI] OR Follow up[TI] OR Interview*[TI] OR Longitudinal[TI] OR Observational[TI] OR Population based[TI] OR Qualitative[TI] OR Questionnaire*[TI] OR Prospective[TI] OR Retrospective[TI] OR Survey[TI] OR Surveys[TI] OR Trend[TI] OR Trends[TI]	Comparative study/or Observational study/or Cohort analysis/or Longitudinal study/or Prospective study/OR Retrospective study/or Cross-sectional study/or Cultural anthropology/or Ethnography/or Empirical Research/or Exp Interview/or Qualitative research/or Exp Questionnaire/or Clinical practice/or Trend study/or ("Before and after" OR Cohort OR Comparison OR Comparative OR Cross sectional OR Cultural anthropolog* OR Descriptive OR Empirical OR Ethnograph* OR Focus group? OR Follow back OR Follow up OR Interview? OR Longitudinal OR Observational OR Population based OR Qualitative OR Questionnaire? OR Prospective OR Retrospective OR Survey? OR Trend?).ti	(Empirical study or Focus group or Followup study or Interview or Longitudinal study or Prospective study or Qualitative study or Quantitative study or Retrospective study).md or Cross Cultural Differences/or "Culture (Anthropological)"/or Ethnography/or Questionnaires/or Exp Surveys/or Clinical practice/or Trends/	TS = ("Before and after" OR Cohort OR "Cross sectional" OR "Cultural anthropolog*" OR Empirical OR Ethnograph* OR "Focus group?" OR "Follow back" OR "Follow up" OR Interview? OR Longitudinal OR Observational OR "Population based" OR Qualitative OR Questionnaire? OR Prospective OR Retrospective OR Survey? OR Trend?) or TI = (Comparison OR Comparative OR Descriptive)	MH "Comparative studies" OR MH "Cross Sectional Studies" OR MH "Prospective studies + " OR MH "Retrospective design" OR MH "Questionnaires + " OR MH "Empirical Research" OR MH "Qualitative Studies + " OR MH "Focus groups" or MH Interviews + OR MH "Quantitative Studies" OR MH "Quasi-Experimental Studies + " OR TI ("Before and after" OR Cohort OR Comparison OR Comparative OR Cross sectional OR Cultural anthropolog* OR Descriptive OR Empirical OR Ethnograph* OR Focus group? OR Follow back OR Follow up OR Interview? OR Longitudinal OR Observational OR Population based OR Qualitative OR Questionnaire? OR Prospective OR Retrospective OR Survey? OR Trend?)				

*truncation symbol to search for all terms that have the same root words

## Appendix 2

**Table 3:** Variations in study methods for 39 jurisdiction-wide surveys and 22 included in analysis of practice variation

Surveys [1]	Included for practice variation analysis	Reasons for exclusion [2]	Article information [3]			Characteristics of professionals surveyed	Characteristics of patients	Methods to identify physicians [4]	Total number of deaths studied or applicable [5]	Clear selection criteria	Data collection method	Practice measurement method	Differential response [6]	Selective reporting [7]	Response rates
			Title	Year of publication	Authors										
Netherlands 1995a	Yes		Euthanasia and other end-of-life decisions in the Netherlands in 1990,1995, and 2001	2003	Onwuteaka-Philipsen, Bregie D.van der Heide, AgnesKoper, DirkKeij-Deerenberg, Ingeborg Rietjens, Judith A.Rurup, Mette L.Vrakking, Astrid M.Georges, Jean JacquesMuller, Martien T.van der Wal, Gerritvan der Maas, Paul J.	1) Specialities; 2) to be actively practising medicine at the time of interview and had to have done so for the previous 2 years in the same specialty and place	Exclusion criteria: the cause of death precluded any kind of end-of-life decision (e.g., a car accident resulting in instant death)	Death certificates	135675	Yes	Self-administered postal questionnaire	Annual incidence	Uncertain	Yes	77.0 %
Netherlands 1990a	Yes		Euthanasia and other medical decisions concerning the end of life	1991	van der Maas, P. J.Vandelden, J. J. M.Pijnenborg, L.Looman, C. W. N.	1) A stratified random sample of 405 physicians was interviewed, including 152 general practitioners, 50 nursing-home physicians, and 203 specialists (cardiologists, surgeons, and specialists in internal medicine, chest disease, and neurology). (Method, section I Interviews with physicians); 2) 6642 Dutch doctors who had signed a death certificate for which medical end-of-life decision were possible (excluding sudden deaths, such accidents.) 5197 responded and returned the questionnaires	For all inhabitants of the Netherlands the cause of death is reported to the Central Bureau of Statistics (CBS). The name of the patient is not mentioned on the cause-of-death form but that of the reporting physician is. The medical officer in charge of the cause-of-death statistics drew a stratified sample of 7000 deaths from Aug 1 to Dec 1, 1990.	Death certificates	128824	Yes	NA	Annual incidence	No	NA	76.0 %
Netherlands 2001a	Yes		Euthanasia and other end-of-life decisions in the Netherlands in 1990,1995, and 2001	2003	Onwuteaka-Philipsen, Bregie D.van der Heide, AgnesKoper, DirkKeij-Deerenberg, Ingeborg Rietjens, Judith A.Rurup, Mette L.Vrakking, Astrid M.Georges, Jean JacquesMuller, Martien T.van der Wal, Gerritvan der Maas, Paul J.	1) Specialities; 2) to be actively practising medicine at the time of interview and had to have done so for the previous 2 years in the same specialty and place.	Exclusion criteria: the cause of death precluded any kind of end-of-life decision (e.g., a car accident resulting in instant death)	Death certificates	140377	Yes	Self-administered postal questionnaire	Annual incidence	Not reported	Yes	74.00 %
Netherlands 1990b	Yes		Euthanasia and other medical decisions concerning the end of life	1991	van der Maas, P. J.Vandelden, J. J. M.Pijnenborg, L.Looman, C. W. N.	1) A stratified random sample of 405 physicians was interviewed, including 152 general practitioners, 50 nursing-home physicians, and 203 specialists (cardiologists, surgeons, and specialists in internal medicine, chest disease, and neurology). (Method, section I Interviews with physicians); 2) 6642 Dutch doctors who had signed a death certificate for which medical end-of-life decision were possible (excluding sudden deaths, such accidents.) 5197 responded and returned the questionnaires (Method; Section II Death certificates)	For all inhabitants of the Netherlands the cause of death is reported to the Central Bureau of Statistics (CBS). The name of the patient is not mentioned on the cause-of-death form but that of the reporting physician is. The medical officer in charge of the cause-of-death statistics drew a stratified sample of 7000 deaths from Aug 1 to Dec 1, 1990.	Physicians	128824	Yes	Interview	Annual incidence	Yes	Yes	68.0 %
Netherlands 2010a	Yes		Trends in end-of-life practices before and after the enactment of the euthanasia law in the Netherlands from 1990 to 2010: a repeated cross-sectional survey	2012	Onwuteaka-Philipsen, Bregje D.Brinkman-Stoppelenburg, AriannePenning, Corinede Jong-Krul, Gwen J.van Delden, Johannes J.van der Heide, Agnes	All attending physicians of the sampled cases in strata two to five (See question 6) received a questionnaire.	1) All deaths that occurred in that period were assigned to one of five strata. When the cause of death clearly precluded end-of-life decision-making), cases were assigned to stratum one. These cases were retained in the sample, but no questionnaires were sent out to the physician. Cases were assigned to one of the other strata looking at the likelihood that an end-of-life decision had preceded death: when this decision was unlikely cause of death was allocated to stratum two, when this decision was possible to stratum three, and when this decision was more probable (e.g., cancer) to stratum four. Cases were assigned to stratum five when the physician had noted on the death certificate that they had actively ended the life of the patient.	Death certificates	136056	Yes	Self-administered postal questionnaire	Annual incidence	Yes	Yes	74.0 %
Belgium (Flanders) 2001/2002a	Yes		End-of-life medical decisions in six European countries: Belgium, Denmark, Italy, the Netherlands, Sweden and Switzerland. [Dutch] medische besluiten rond het levenseinde in 6 europese landen: belgie, denemarken, italie, nederland, zweden en zwitserland	2003	Van Der Heide, A.Deliens, L.Faisst, K.Nilstun, T.Norup, M.Paci, E.Van Der Wal, G.Van Der Maas, P. J.	Danish, Italian, and Dutch samples included a stratum in which end-of-life decisions were precluded on the basis of information on the death certificate, for which no questionnaires were sent out. For all other sampled cases, the attending doctors were asked if death had arisen suddenly and unexpectedly.	Random samples of death certificates of people aged 1 year or older from death registries to which all deaths are reported. The sampling period varied between 3 and 6 months, but all deaths that we included arose between June 2001, and February 2002.	EURELD-death certificates	55793	Yes	NA	Annual incidence	No	Yes	59.0 %
Netherlands 2001b	Yes		Euthanasia and other end-of-life decisions in the Netherlands in 1990, 1995, and 2001	2003	Onwuteaka-Philipsen, Bregie D.van der Heide, AgnesKoper, DirkKeij-Deerenberg, IngeborgRietjens, Judith A.Rurup, Mette L.Vrakking, Astrid M.Georges, Jean JacquesMuller, Martien T.van der Wal, Gerritvan der Maas, Paul J.	1) Specialities; 2) to be actively practising medicine at the time of interview and had to have done so for the previous 2 years in the same specialty and place	Exclusion criteria: the cause of death precluded any kind of end-of-life decision (e.g., a car accident resulting in instant death)	Physicians	140377	Yes	Interview	Annual incidence	Yes	Yes	85.0 %
Denmark 2001/2002a	Yes		End-of-life medical decisions in six European countries: Belgium, Denmark, Italy, the Netherlands, Sweden and Switzerland. [Dutch] medische besluiten rond het levenseinde in 6 europese landen: belgie, denemarken, italie, nederland, zweden en zwitserland	2003	Van Der Heide, A.Deliens, L.Faisst, K.Nilstun, T.Norup, M.Paci, E.Van Der Wal, G.Van Der Maas, P. J.	Danish, Italian, and Dutch samples included a stratum in which end-of-life decisions were precluded on the basis of information on the death certificate, for which no questionnaires were sent out. For all other sampled cases, the attending doctors were asked if death had arisen suddenly and unexpectedly.	Random samples of death certificates of people aged 1 year or older from death registries to which all deaths are reported. The sampling period varied between 3 and 6 months, but all deaths that we included arose between June 2001, and February 2002.	EURELD-death certificates	58722	Yes	NA	Annual incidence	No	Yes	62.0 %
Netherlands 1994–1998 (ALS patients)	No	Specific population of patients	Euthanasia and physician-assisted suicide in Dutch patients with amyotrophic lateral sclerosis. [Dutch] euthanasie en hulp bij zelfdoding bij patienten met amyotrofische laterale sclerose in nederland	2004	Veldink, J. H.Wokke, J. H. J.Van Der Wal, G.De Jong, J. M. B. V.Van Den Berg, L. H.	The family physicians of 279 Dutch patients who fulfilled the criteria for possible, probable or definite ALS, who were known in the Utrecht University Medical Centre or the Academic Medical Centre in Amsterdam, the Netherlands, and who had died in the period 1994–1998 were asked to fill out a validated questionnaire about the various medical end-of-life decisions that had been taken and their possible clinical, care-related and social determinants.	NA	ALS patients	279	Yes	Self-administered postal questionnaire	Annual incidence	No	Yes	84.0 %
Australia 1996b	Yes		End-of-life decisions in Australian medical practice	1997	Kuhse, H.Singer, P.Baume, P.Clark, M.Rickard, M.	1. 3000 doctors taken at random from a list of 27000 Australian doctors extracted from the Australian Medical Masterfile Database (Australasian Medical Publishing Company, Sydney), one of 27 medical disciplines where there would be the possibility of making a medical end-of-life decision; 2- the 27 medical disciplines (also extracted from the Australian Medical Masterfile Database) as comparable as possible with the broader categories of doctors (cardiology, surgery, internal medicine, respiratory medicine [pulmonology], neurology, general practitioners and nursing home physicians) who were attendant to 87 % of hospital deaths and nearly all deaths outside hospitals in the Netherlands; 3- The initial questions on the questionnaire narrowed the field of respondents to include the 1361 doctors who had attended a death within the last 12 months.	NA	Physicians	125771	Yes	Self-administered postal questionnaire	Annual incidence	Yes	Yes	64.0 %
Sweden 2001/2002a	Yes		End-of-life medical decisions in six European countries: Belgium, Denmark, Italy, the Netherlands, Sweden and Switzerland. [Dutch] medische besluiten rond het levenseinde in 6 europese landen: belgie, denemarken, italie, nederland, zweden en zwitserland	2003	Van Der Heide, A.Deliens, L.Faisst, K.Nilstun, T.Norup, M.Paci, E.Van Der Wal, G.Van Der Maas, P. J.	Danish, Italian, and Dutch samples included a stratum in which end-of-life decisions were precluded on the basis of information on the death certificate, for which no questionnaires were sent out. For all other sampled cases, the attending doctors were asked if death had arisen suddenly and unexpectedly.	Random samples of death certificates of people aged 1 year or older from death registries to which all deaths are reported. The sampling period varied between 3 and 6 months, but all deaths that we included arose between June 2001, and February 2002.	EURELD-death certificates	93755	Yes	NA	Annual incidence	No	Yes	61.0 %
Netherlands 2001/2002a	Yes		End-of-life medical decisions in six European countries: Belgium, Denmark, Italy, the Netherlands, Sweden and Switzerland. [Dutch] medische besluiten rond het levenseinde in 6 europese landen: belgie, denemarken, italie, nederland, zweden en zwitserland	2003	Van Der Heide, A.Deliens, L.Faisst, K.Nilstun, T.Norup, M.Paci, E.Van Der Wal, G.Van Der Maas, P. J.	Danish, Italian, and Dutch samples included a stratum in which end-of-life decisions were precluded on the basis of information on the death certificate, for which no questionnaires were sent out. For all other sampled cases, the attending doctors were asked if death had arisen suddenly and unexpectedly.	Random samples of death certificates of people aged 1 year or older from death registries to which all deaths are reported. The sampling period varied between 3 and 6 months, but all deaths that we included arose between June 2001, and February 2002.	EURELD-death certificates	140377	Yes	NA	Annual incidence	No	Yes	75.0 %
Switzerland (German- speaking) 2001/2002a	Yes		End-of-life medical decisions in six European countries: Belgium, Denmark, Italy, the Netherlands, Sweden and Switzerland. [Dutch] medische besluiten rond het levenseinde in 6 europese landen: belgie, denemarken, italie, nederland, zweden en zwitserland	2003	Van Der Heide, A.Deliens, L.Faisst, K.Nilstun, T.Norup, M.Paci, E.Van Der Wal, G.Van Der Maas, P. J.	Danish, Italian, and Dutch samples included a stratum in which end-of-life decisions were precluded on the basis of information on the death certificate, for which no questionnaires were sent out. For all other sampled cases, the attending doctors were asked if death had arisen suddenly and unexpectedly.	Random samples of death certificates of people aged 1 year or older from death registries to which all deaths are reported. The sampling period varied between 3 and 6 months, but all deaths that we included arose between June 2001, and February 2002.	EURELD-death certificates	44036	Yes	NA	Annual incidence	No	Yes	67.0 %
UK 2007/2008b	Yes		Hastening death in end-of-life care: a survey of doctors	2009	Seale, Clive	1) Binley’s database (http://www.binleys.com/) of UK medical practitioners was used to send questionnaires to 8857 working UK medical practitioners, comprising separate random samples of 2829 GPs, 443 neurologists, 836 specialists in care of the elderly, 462 specialists in palliative medicine and 4287 in other hospital specialties in 2007 to 2008; 2) Excluding specialties such as public health where doctors do not normally treat people who die; 3) Neurologists, palliative medicine and care of the elderly specialists were over sampled in relation to their proportions in the medical population to enable exploration of the circumstances of elderly people, people receiving specialist palliative care, and those with multiple sclerosis (MS) and motor neurone disease (MND).	NA	Physicians	72071	Yes	Self-administered postal questionnaire	Annual incidence	Yes	Yes	42.1 %
Italy 2001/2002a	Yes		End-of-life medical decisions in six European countries: Belgium, Denmark, Italy, the Netherlands, Sweden and Switzerland. [Dutch] medische besluiten rond het levenseinde in 6 europese landen: belgie, denemarken, italie, nederland, zweden en zwitserland	2003	Van Der Heide, A.Deliens, L.Faisst, K.Nilstun, T.Norup, M.Paci, E.Van Der Wal, G.Van Der Maas, P. J.	Danish, Italian, and Dutch samples included a stratum in which end-of-life decisions were precluded on the basis of information on the death certificate, for which no questionnaires were sent out. For all other sampled cases, the attending doctors were asked if death had arisen suddenly and unexpectedly.	Random samples of death certificates of people aged 1 year or older from death registries to which all deaths are reported. The sampling period varied between 3 and 6 months, but all deaths that we included arose between June 2001, and February 2002.	EURELD-death certificates	22368	Yes	NA	Annual incidence	No	Yes	44.0 %
Belgium (Flanders) 1998a	Yes		The incidence and characteristics of end-of-life decisions by GPs in Belgium	2004	Bilsen, JohanStichele, Robert VanderMortier, FreddyBernheim, JanDeliens, Luc	Identified the speciality of the attesting physicians (GP or specialist) for each of the 3999 sampled death certificates and selected only the cases in the data set for which a GP returned the questionnaire	"A 20 % random sample was taken from all death certificates signed between January 1 and April 30, 1998."	Death certificates	56354	Yes	Self-administered postal questionnaire	Annual incidence	Not reported	No	64.8 %
Belgium 2005/2006a	Yes		Euthanasia and other end of life decisions and care provided in final three months of life: nationwide retrospective study in Belgium	2009	Van Den Block, L.Deschepper, R.Bilsen, J.Bossuyt, N.Van Casteren, V.Deliens, L.	Inclusion criteria: registered the death of a patient between 1 January 2005 and 31 December 2006 in Belgium	Inclusion Criteria: 1) Died between 1 January 2005 and 31 December 2006 in Belgium; 2) Be over the age of one year; 3) Non-sudden death	Death certificates	2690	Yes	NA	Annual incidence	Yes	Yes	64.3 %
France 2009a	Yes		End-of-life medical decisions in France: a death certificate follow-up survey 5 years after the 2005 act of parliament on patients' rights and end of life	2012	Pennec, S.Monnier, A.Pontone, S.Aubry, R.	Inclusion criteria: Having completed a death certificate in December 2009	1) Aged 18 and over; 2) died in France in December 2009	Death certificates	47872	Yes	Self-administered postal questionnaire	Annual incidence	Not reported	Yes	40.0 %
Netherlands 1990 (Prospective)	Yes		Euthanasia and other medical decisions concerning the end of life	1991	van der Maas, P. J.Vandelden, J. J. M.Pijnenborg, L.Looman, C. W. N.	1) A stratified random sample of 405 physicians was interviewed, including 152 general practitioners, 50 nursing-home physicians, and 203 specialists (cardiologists, surgeons, and specialists in internal medicine, chest disease, and neurology). (Method, section I Interviews with physicians); 2) 6642 Dutch doctors who had signed a death certificate for which medical end-of-life decision were possible (excluding sudden deaths, such accidents.) 5197 responded and returned the questionnaires	For all inhabitants of the Netherlands the cause of death is reported to the Central Bureau of Statistics (CBS). The name of the patient is not mentioned on the cause-of-death form but that of the reporting physician is. The medical officer in charge of the cause-of-death statistics drew a stratified sample of 7000 deaths from Aug 1 to Dec 1, 1990.	Prospective	128786	Yes	Self-administered postal questionnaire	Annual incidence	Not reported	Yes	80.0 %
Netherlands 1995b	Yes		Euthanasia and other end-of-life decisions in the Netherlands in 1990,1995, and 2001	2003	Onwuteaka-Philipsen, Bregie D.van der Heide, AgnesKoper, DirkKeij-Deerenberg, IngeborgRietjens, Judith A.Rurup, Mette L.Vrakking, Astrid M.Georges, Jean JacquesMuller, Martien T.van der Wal, Gerritvan der Maas, Paul J.	1) Specialities; 2) to be actively practising medicine at the time of interview and had to have done so for the previous 2 years in the same specialty and place	Exclusion criteria : the cause of death precluded any kind of end-of-life decision (e.g., a car accident resulting in instant death)	Physicians	135675	Yes	Interview	Annual incidence	Not reported	Yes	74.0 %
Belgium (Flanders) 1999/2000c	No	Specific population of patients	The first five years of euthanasia legislation in Belgium and the Netherlands: Description and comparison of cases	2012	Rurup, Mette L.Smets, TinneCohen, JoachimBilsen, JohanOnwuteaka-Philipsen, Bregje D.Deliens, Luc	The anonymized databases of the reported cases of euthanasia in Belgium and the Netherlands were made available by the review committees. We selected all cases reported between 22 September 2002 (date of first report in Belgium) and the end of 2007 in both databases	The anonymized databases of the reported cases of euthanasia in Belgium and the Netherlands were made available by the review committees. We selected all cases reported between 22 September 2002 (date of first report in Belgium) and the end of 2007 in both databases	Neonates/infants	292	Yes	Self-administered postal questionnaire	Annual incidence	Not reported	Yes	87.0 %
Netherlands 2005a	Yes		End-of-Life Practices in the Netherlands under the Euthanasia Act	2007	van der Heide, A., B. D. Onwuteaka-Philipsen, M. L. Rurup, H. M. Buiting, J. J. van Delden, J. E. Hanssen-de Wolf, A. G. Janssen, H. Pasman, J. A. Rietjens, C. J. Prins, I. M. Deerenberg, J. K. Gevers, P. J. van der Maas and G. van der Wal	1) To be licensed physicians practicing in Oregon from the Oregon State Board of Medical Examiners (BME) in November 1994; 2) We defined “attending physicians” as all physicians in Oregon licensed in the following specialties: internal medicine, family practice, general practice, neurology, gynaecology, therapeutic radiology, and surgery	NA	Death certificates	136402	Yes	Self-administered postal questionnaire	Annual incidence	Not reported	Yes	77.80 %
Netherlands 2001c	No	Specific population of patients	[No conspicuous changes in the practice of medical end-of-life decision-making for neonates and infants in the Netherlands in 2001 as compared to 1995]	2005	Vrakking, A. M.van der Heide, A.Onwuteaka-Philipsen, B. D.Keij-Deerenberg, I. M.van der Maas, P. J.van der Wal, G.	The questionnaires which were sent to the physicians who reported the deaths, included structured questions about whether or not death had been preceded by end-of-life decisions, i.e. decisions to withhold or withdraw potentially life-prolonging treatment or to administer (potentially) life-shortening drugs, and questions about the decision-making process.	In both years, all deaths of children under the age of one year that took place in August-November (1995: *n* = 338; 2001: *n* = 347) were studied	Neonates/infants	1088	Yes	Self-administered postal questionnaire	Annual incidence	Not reported	Yes	84.0 %
Netherlands 2005/2006 (HGG patients)	No	Specific population of patients	Decision-making in the end-of-life phase of high-grade glioma patients	2012	Sizoo, E. M.Pasman, H. R.Buttolo, J.Heimans, J. J.Klein, M.Deliens, L.Reijneveld, J. C.Taphoorn, M. J.	"The physicians involved in end-of-life care of deceased patients of the cohort were approached for participation in the study. (…) If more than one physician was involved in end of life care for a specific patient (for example due to a transition in health care setting close before death), all physicians were approached for participation in the study"	"adult HGG patients diagnosed in 2005 and 2006 in three tertiary referral centres for brain tumour patients (VU University Medical Centre and Academic Medical Centre Amsterdam Amsterdam, Medical Centre Haaglanden The Hague, The Netherlands)"	HGG patients	223	Yes	Self-administered questionnaire	Annual incidence	No	Yes	62.0 %
Netherlands 1995c	No	Specific population of patients	[No conspicuous changes in the practice of medical end-of-life decision-making for neonates and infants in the Netherlands in 2001 as compared to 1995]	2005	Vrakking, A. M.van der Heide, A.Onwuteaka-Philipsen, B. D.Keij-Deerenberg, I. M.van der Maas, P. J.van der Wal, G.	The questionnaires which were sent to the physicians who reported the deaths, included structured questions about whether or not death had been preceded by end-of-life decisions, i.e. decisions to withhold or withdraw potentially life-prolonging treatment or to administer (potentially) life-shortening drugs, and questions about the decision-making process.	In both years, all deaths of children under the age of one year that took place in August-November (1995: *n* = 338; 2001: *n* = 347) were studied	Neonates/infants	1041	Yes	Self-administered postal questionnaire	Annual incidence	Not reported	Yes	88.0 %
Belgium (Hasselt, Flanders) 1996a	Yes		Attitudes, socio-demographic characteristics, and actual end-of-life decisions of physicians in Flanders, Belgium	2003	Mortier, F.Bilsen, J.Vander Stichele, R. H.Bernheim, J.Deliens, L.	"All physicians who signed a death certificate" in 1996 in the city of Hasselt (Flanders)	"All official death certificates of the 970 deaths in Hasselt in 1996 were retrieved for the study."	Death certificates	970	Yes	Self-administered postal questionnaire	Annual incidence	Yes	Yes	55.0 %
Belgium (Flanders) 2007a	Yes		Trends in medical end-of-life decision making in Flanders, Belgium 1998-2001-2007	2011	Chambaere, K.Bilsen, J.Cohen, J.Onwuteaka-Philipsen, B. D.Mortier, F.Deliens, L.	Every certifying physician was sent a 5-page questionnaire for a maximum of 5 cases, with at most 3 reminders in case of nonresponse.	We performed a death certificate survey in Flanders, the Flemish-speaking part of Belgium, which has about 6 million inhabitants and approximately 55,000 deaths per year. This study was similar to those performed in 1998 and 2001. A stratified random sample of deaths was drawn by the central administration authority for death certificates, the Flemish Agency for Care and Health. All deaths between 1 June 2007 and 30 November 2007 of Belgian residents aged 1 year or older were first assigned to 1 of 4 strata, based on the underlying cause of death as indicated on the death certificate and the estimated corresponding likelihood of an end-of-life practice. Sampling fractions for each stratum increased with this likelihood.	Death certificates	54881	Yes	Self-administered postal questionnaire	Annual incidence	Not reported	Yes	58.4 %
Netherlands 2005/2006 (Neonates/infants (Aug to Nov))	No	Specific population of patients	Analgesics, sedatives and neuromuscular blockers as part of end-of-life decisions in Dutch NICUs	2009	Verhagen, A. A.Dorscheidt, J. H. Engels, B.Hubben, J. H.Sauer, P. J.	Caring of a infants for newborns in group II (Patients and methods - Interviews, p.F435); Group II : stabilised newborns with a poor prognosis	"infants who died before the age of 2 months between October 2005 and September 2006 in the NICUs"	Neonates/infants (Aug to Nov)	NA	Yes	Interview	Annual incidence	Not reported	Yes	97.80 %
Sweden 1998b	No	Specific population of physicians	Palliative care, assisted suicide and euthanasia: Nationwide questionnaire to Swedish physicians	2000	Valverius, E.Nilstun, T.Nilsson, B.	Inclusion criteria: Swedish Pharmaceutical Statistics OR working in palliative care units in Sweden OR members of the Swedish Association for the Study of Pain	Inclusion criteria: deceased during 1997	Physicians	952	Yes	Self-administered postal questionnaire	Annual incidence	No	Yes	78.0 %
UK 2004b	Yes		National survey of end-of-life decisions made by UK medical practitioners	2006	Seale, C.	1) A random sample of 1000 general practitioners (GPs) and 1000 hospital specialists listed on Binley’s database (www.binleys.com) of all working UK medical practitioners (updated in September 2004) were sent questionnaires, with two follow-up reminders, between October and December 2004; 2) specialties where doctors could not be expected to have attended a death in the previous year (e.g., public health) were excluded	NA	Physicians	22558	Yes	Self-administered postal questionnaire	Annual incidence	Not reported	Yes	53.0 %
Netherlands 2005/2006 (Neonates/infants (group I))	No	Specific population of patients	Analgesics, sedatives and neuromuscular blockers as part of end-of-life decisions in Dutch NICUs	2009	Verhagen, A. A.Dorscheidt, J. H.Engels, B.Hubben, J. H.Sauer, P. J.	Caring of a infants for newborns in group II (Patients and methods - Interviews, p.F435); Group II : stabilised newborns with a poor prognosis	"infants who died before the age of 2 months between October 2005 and September 2006 in the NICUs"	Neonates/infants (group I)	359	Yes	NA	Annual incidence	Yes	Yes	98.00 %
Belgium (Flanders) 2007 (Death certificates (cancer patients))	Yes		Trends in End-of-Life Decision Making in Patients With and Without Cancer	2013	Pardon, KoenChambaere, KennethPasman, H. Roeline W.Deschepper, ReginaldRietjens, JudithDeliens, Luc	Physicians who had attested to the sampled death certificates were sent a 5-page paper-and-pencil questionnaire by the Flemish Agency about the medical decisions made at the patient’s end of life, the decision-making process, and the care provided.	We conducted a nationwide death certificate study in 2007 in Flanders, analogous to our death certificate study of 1998. The Flemish Agency for Care and Health selected a random stratified sample of all death certificates of persons ages 1 year or older from June to November 2007.	Death certificates (cancer patients)	15257	Yes	Self-administered postal questionnaire	Annual incidence	Yes	Yes	58.4 %
Belgium (Flanders) 2007 (Death certificates (non-cancer patients))	Yes		Trends in End-of-Life Decision Making in Patients With and Without Cancer	2013	Pardon, KoenChambaere, KennethPasman, H. Roeline W.Deschepper, ReginaldRietjens, JudithDeliens, Luc	Physicians who had attested to the sampled death certificates were sent a 5-page paper-and-pencil questionnaire by the Flemish Agency about the medical decisions made at the patient’s end of life, the decision-making process, and the care provided.	We conducted a nationwide death certificate study in 2007 in Flanders, analogous to our death certificate study of 1998. The Flemish Agency for Care and Health selected a random stratified sample of all death certificates of persons ages 1 year or older from June to November 2007.	Death certificates (non-cancer patients)	39624	Yes	Self-administered questionnaire	Annual incidence	Yes	Yes	58.4 %
Netherlands 2001 (Children (1 to 17y) (Aug to Dec))	No	Specific population of patients	Medical end-of-life decisions for children in the Netherlands	2005	Vrakking, A. M.van der Heide, A.Arts, W. F.Pieters, R.van der Voort, E.Rietjens, J. A.Onwuteaka-Philipsen, B. D.van der Maas, P. J.van der Wal, G.	Inclusion criteria: have reported a death of a child between August 1 and December 1, 2001.	STUDY 1:Inclusion criteria: have died between August 1 and December 1, 2001 in the Netherlands, aged between 1–17 years	Children (1 to 17y) (Aug to Dec)	610	Yes	Self-administered postal questionnaire	Annual incidence	Not reported	Yes	75.0 %
Netherlands 2005/2006 (Neonates/infants (group II))	No	Specific population of patients	Analgesics, sedatives and neuromuscular blockers as part of end-of-life decisions in Dutch NICUs	2009	Verhagen, A. A.Dorscheidt, J. H.Engels, B.Hubben, J. H.Sauer, P. J.	Caring of a infants for newborns in group II (Patients and methods - Interviews, p.F435); Group II : stabilised newborns with a poor prognosis	"infants who died before the age of 2 months between October 2005 and September 2006 in the NICUs"	Neonates/infants (group II)	359	Yes	NA	Annual incidence	Yes	Yes	98.00 %
Netherlands 1996–1998 (Dementia in nursing homes)	No	Specific population of patients	End-of-Life Decision Making in Nursing Home Residents with Dementia and Pneumonia: Dutch Physicians' Intentions Regarding Hastening Death	2005	van der Steen, Jenny T.van der Wal, GerritMehr, David R.Ooms, Marcel E.Ribbe, Miel W.	Nursing home physicians, who are employed by the nursing home in the Netherlands, completed questionnaires regarding their decisions and treatments at the time of deciding to withhold antibiotics.	We identified eligible subjects from a nationwide Dutch study of 706 nursing home residents with dementia who were diagnosed with pneumonia. For the present analyses, we first selected the 165 (23 %) patients who physicians decided not to treat with antibiotics. As shown in Figure 1, we excluded 22 patients, including 12 who survived for 3 months and 8 who died of another cause or a second episode of pneumonia.	Dementia in nursing homes	143	Yes	Self-administered questionnaire	Annual incidence	No	Yes	86.7 %
Belgium (Flanders) 2007/2008 (NSCLC patients)	No	Specific population of patients	Expressed wishes and incidence of euthanasia in advanced lung cancer patients	2012	Pardon, K.Deschepper, R.Vander Stichele, R.Bernheim, J. L.Mortier, F.Schallier, D.Germonpre, P.Galdermans, D.Van Kerckhoven, W.Deliens, L.Eolic Consortium	We asked the pulmonologist or oncologist and the general practitioner (GP) of the patient to fill in an after-death questionnaire for those patients who died within 18 months of inclusion in the study.	Patients conformed to the following inclusion criteria: a recent initial diagnosis of non-small cell lung cancer (NSCLC) stage IIIb or IV, 18 yrs. or older, Dutch speaking and physically and psychologically able to participate in the study. The patients were recruited consecutively during one year by pulmonologists and oncologists in 13 hospitals in Flanders.	NSCLC patients	291	Yes	Self-administered questionnaire	Annual incidence	Not reported	Yes	91.3 %
Sweden 1998 (Palliative care physicians)	No	Specific population of physicians	Palliative care, assisted suicide and euthanasia: Nationwide questionnaire to Swedish physicians	2000	Valverius, E.Nilstun, T.Nilsson, B.	Inclusion criteria: Swedish Pharmaceutical Statistics OR working in palliative care units in Sweden OR members of the Swedish Association for the Study of Pain	Inclusion criteria: deceased during 1997	Palliative care physicians	122	Yes	Self-administered postal questionnaire	Annual incidence	No	Yes	83.0 %
Sweden 1998 (Association for the Study of Pain Physicians)	No	Specific population of physicians	Palliative care, assisted suicide and euthanasia: Nationwide questionnaire to Swedish physicians	2000	Valverius, E.Nilstun, T.Nilsson, B.	Inclusion criteria: Swedish Pharmaceutical Statistics OR working in palliative care units in Sweden OR members of the Swedish Association for the Study of Pain	Inclusion criteria: deceased during 1997	Association for the Study of Pain Physicians	130	Yes	Self-administered postal questionnaire	Annual incidence	No	Yes	82.0 %
EUROPE 2005 (RICU patients)	No	Specific population of patients	End-of-life decision-making in respiratory intermediate care units: A European survey	2007	Nava, S.Sturani, C.Hartl, S.Magni, G.Ciontu, M.Corrado, A.Simonds, A.	Once approved by the ERS office, a formal letter was sent by e-mail to all of the participants in the census on the epidemiology of RICUs in Europe, performed in 2002, and all members of the ERS Respiratory Intensive Care Assembly to invite them to participate in the present study.	The aim of this task force, conducted between May 1, 2005 and October 31, 2005, was to collect data regarding end-of-life decisions in RICUs and high dependency units (HDUs) within Europe by means of a prospective questionnaire.	RICU patients	6008	Yes	Self-administered web questionnaire	Annual incidence	Yes	Yes	21.5 %
New Zealand 2000b	Yes	Specific population of physicians	End of life decision-making by New Zealand general practitioners: A national survey	1196	Mitchell, K.Owens, R. G.	1) The questionnaire was administered to GPs in New Zealand (in August and September, 2000). It asked for details on the last death in the previous 12 months for which the physician was the attendant doctor, and whether that physician had access to a multidisciplinary palliative care team; 2) There are approximately 3000 practising GPs in New Zealand and a questionnaire was sent to 2602 on a commercial mailing list.	NA	Physicians	693	Yes	Self-administered postal questionnaire	Annual incidence	Uncertain	Yes	48.0 %

*Note*: [1] the end-of-life surveys were identified by the jurisdictions of investigation, year of study and methods to identify physicians; a = death certificate studies, b = physician survey, c = neonate or infant study; [2] the term “exclusion” meant the surveys excluded from statistical analyses with other surveys because of the focus on specific populations or geographic regions; [3] the article listed in the table was the one that used for data extraction; [4] the methods to identify physicians included death certificates, physicians (registries or associations), neonate/infants (patients or death certificates), or other specific patient groups; [5] the numbers in the column might be the total numbers of deaths that the frequencies of end-of-life practices could be applied or total numbers of death studied; [6] differential response meant the response rates might differ across sampling strata or subgroups; [7] selective reporting meant that not all results of a single study were reported in the article

## Appendix 3

**Table 4:** Coefficients of logit regression models for the frequencies of end-of-life practices

	1. Withholding or withdrawal of treatment				2.1 Use of opioids with possible life shortening effects				2.2 Use of sedatives with possible life shortening effects				3.1 Intentional use of lethal drugs self-dministered by patients under patient request				3.2 Intentional use of lethal drugs, administered by professionals under patient request				3.3 Intentional use of lethal drugs administered by professionals without patient voluntary request			
		(95 % CI)				(95 % CI)				(95 % CI)				(95 % CI)				(95 % CI)				(95 % CI)		
Variables	Coefficients	(2.5 %	97.5 %)	*p*	Coefficients	(2.5 %	97.5 %)	*p*	Coefficients	(2.5 %	97.5 %)	*p*	Coefficients	(2.5 %	97.5 %)	*p*	Coefficients	(2.5 %	97.5 %)	*p*	Coefficients	(2.5 %	97.5 %)	*p*
Year	−0.002	−0.003	−0.002	<0.001	0.044	0.043	0.045	<0.001	0.101	0.098	0.104	<0.001	−0.049	−0.056	−0.042	<0.001	0.016	0.014	0.018	<0.001	−0.057	−0.061	−0.053	<0.001
Survey types																								
Death certificates	(reference)																							
Physicians	0.94	0.92	0.95	<0.001	0.02	0.01	0.04	0.01	0.58	0.55	0.60	<0.001	0.25	0.16	0.33	<0.001	0.00	−0.03	0.03	1.00	0.07	0.01	0.12	0.02
Prospective	−0.14	−0.16	−0.12	<0.001	−0.17	−0.19	−0.15	<0.001					0.42	0.31	0.54	<0.001	0.29	0.24	0.33	<0.001	0.54	0.48	0.60	<0.001
Jurisdictions																								
Netherlands	(reference)																							
Australia	−0.39	−0.41	−0.37	<0.001	0.59	0.57	0.61	<0.001					−0.94	−1.13	−0.75	<0.001	−0.18	−0.22	−0.13	<0.001	1.59	1.54	1.65	<0.001
Belgium	−0.20	−0.33	−0.06	<0.001	0.00	−0.11	0.11	0.98	0.13	−0.04	0.29	0.13									1.36	0.97	1.76	<0.001
Belgium (Flanders)	−0.19	−0.20	−0.17	<0.001	−0.10	−0.11	−0.09	<0.001	0.44	0.42	0.46	<0.001	−0.17	−0.31	−0.02	0.03	−0.72	−0.77	−0.67	<0.001	1.59	1.55	1.64	<0.001
Denmark	−0.34	−0.37	−0.32	<0.001	0.13	0.11	0.15	<0.001	−0.86	−0.92	−0.81	<0.001	−0.94	−1.28	−0.60	<0.001	−3.69	−4.03	−3.35	<0.001	0.29	0.19	0.40	<0.001
France	0.03	0.00	0.05	0.03	−0.09	−0.12	−0.07	<0.001																
Italy	−1.71	−1.77	−1.64	<0.001	−0.27	−0.31	−0.24	<0.001	0.42	0.37	0.47	<0.001	−15.08	−406.00	375.83	0.94	−4.08	−4.75	−3.42	<0.001	−2.16	−2.71	−1.60	<0.001
Sweden	−0.34	−0.36	−0.32	<0.001	−0.15	−0.17	−0.13	<0.001	−0.61	−0.65	−0.57	<0.001	−15.08	−206.02	175.86	0.87	−14.83	−57.44	27.77	0.49	−0.78	−0.92	−0.64	<0.001
Switzerland (German- speaking)	0.53	0.51	0.55	<0.001	−0.09	−0.11	−0.07	<0.001	−0.19	−0.24	−0.14	<0.001	0.86	0.69	1.03	<0.001	−2.18	−2.36	−1.99	<0.001	−0.17	−0.33	−0.02	0.02
UK	3.28	1.57	4.99	<0.001	−0.41	−0.44	−0.38	<0.001	−204.15	−209.97	−198.32	<0.001	−15.08	−205.07	174.91	0.87	−2.57	−2.72	−2.42	<0.001	−0.26	−0.40	−0.13	<0.001

## Abbreviations

CI: Confidence interval
CINAHL: Cumulative index to nursing and allied health literature
EMBASE: Excerpta Medica dataBASE
EURELD: European End-of-life Decisions Consortium
MEDLINE: Medical literature analysis and retrieval system online
PAIS: Public affairs information service
SCI: Science citation index
SSCI: Social sciences citation index
